# Exosomal miRNA‐1231 derived from bone marrow mesenchymal stem cells inhibits the activity of pancreatic cancer

**DOI:** 10.1002/cam4.2633

**Published:** 2019-10-23

**Authors:** Song Shang, Jinfeng Wang, Shilin Chen, Renyun Tian, Hui Zeng, Liang Wang, Man Xia, Haizhen Zhu, Chaohui Zuo

**Affiliations:** ^1^ Department of Gastroduodenal and Pancreatic Surgery Translational Medicine Research Center of Liver Cancer Laboratory of Digestive Oncology The Affiliated Cancer Hospital of Xiangya School of Medicine Central South University Hunan Cancer Hospital Changsha China; ^2^ Department of Molecular Medicine College of Biology State Key Laboratory of Chemo/Biosensing and Chemometrics Hunan University Changsha China; ^3^ Graduates School University of South China Hengyang China; ^4^ Department of Gynecological Oncology The Affiliated Cancer Hospital of Xiangya School of Medicine Central South University Hunan Cancer Hospital Changsha China

**Keywords:** BM‐MSCs, exosomes, miR‐1231, oncogenic activity, pancreatic cancer

## Abstract

Pancreatic cancer (PC) is a highly malignant tumor with increased morbidity and mortality, which is difficult to diagnose and cure in the clinic. Through secreting exosomes containing biological molecules, including diverse RNAs and proteins, bone marrow mesenchymal stem cells (BM‐MSCs) influence the immunity, inflammation, tumor environment, and cancer metastasis. In this study, low expression of miRNA‐1231 (miR‐1231) in exosomes derived from the peripheral blood was significantly correlated with the TNM stage of PC, suggesting the potential inhibitory effect of exosomal miR‐1231 on PC occurrence and development. The proliferation, migration, invasion, and adhesion to the matrix of PC cells BxPC‐3 and PANC‐1 were negatively regulated by exosomes derived from the supernatants of BM‐MSCs that transfected with miR‐1231 oligonucleotides. Simultaneously, tumor growth in vivo was seriously restrained in BALB/C nude mice by tail vein injection with exosomes originated from BM‐MSCs that transfected with miR‐1231 mimics. The exosomes extracted from BM‐MSCs with high level of miR‐1231 inhibit the activity of PC, providing the potential application for developing new and efficient medicine for cancer therapy, especially for PC treatment. The exosomal miR‐1231 of peripheral blood may also be a potential indicator for PC diagnosis in the future.

## Graphical abstract

The expression change of exosomal miR‐1231 derived from BM‐MSCs may provide important reference value for cancer diagnosis, especially for pancreatic cancer. And the exosomes with a high level of miR‐1231 may develop as promising new and highly efficient medicines for pancreatic cancer therapy.

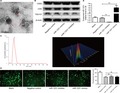

## 1 INTRODUCTION

Malignant tumor is a worldwide disease with high morbidity and mortality. And nearly half of the new diagnosed cancers appeared in China. Among them, the pancreatic cancer (PC), as one of the top ten malignant tumors in China, is characterized with extremely high malignancy, rapid progression, low rate of early diagnosis, and radical resection.^1^ The 5‐year survival of PC is only about 6%.^2^ According to previous reports, 80% of PC patients are diagnosed at the middle and late stage, and nearly 50% of cases are universally found with liver metastasis.^3^ Therefore, it is mandatory to uncover more efficient solutions for cancer therapy in the clinic, especially for PC. Since nearly 90% of PC cases are diagnosed as pancreatic ductal adenocarcinoma (PDA), our study of PC was focused on PDA research.

Stem cells originate from bone marrow, which includes hematopoietic stem cells (HSCs), bone marrow mesenchymal stem cells (BM‐MSCs), and bone marrow hematopoietic progenitor cells (BM‐HPCs), which are labeled with self‐renewal, differentiation, dormancy, and hematopoiesis. Cytokines and extracellular matrix secreted from BM‐HPCs form a niche for supporting the metastasis of carcinoma in situ and the growth of metastases.^4^ BM‐MSCs promote the invasiveness and migration of osteosarcoma and hepatic cancer.^5^, ^6^ However, BM‐MSCs promote colorectal cancer cell death under low‐dose irradiation.^7^ And exosomes derived from siRNA against GRP78 modified BM‐MSCs suppress Sorafenib resistance in hepatocellular carcinoma.^8^ So no defined function of BM‐MSCs in cancer has been concluded up to now. In all, BM‐MSCs are key players in constituting the tumor microenvironment.^9^

Extracellular vesicles come in multiple forms.^10^ Exosomes, derived from the endocytic pathway, are a kind of membranous vesicle with a diameter about 40‐200 nm, saucer shape under electron microscope, and special expression of markers such as CD9, CD63, and TSG101.^11^, ^12^ Exosomes carry bioactive molecules, influencing the extracellular environment and cancer biology to a great extent.^13^, ^14^ For example, exosomal PD‐L1 enhances the immune evasion of cancer cells, resulting in the resistance of PD‐1/PD‐L1 blockades.^15^ However, suppressing exosomal PD‐L1 induces systemic anti‐tumor immunity and memory.^16^ Interestingly, BM‐MSCs‐derived exosomes facilitate multiple myeloma progression.^17^ Exosomes, as nontoxic and nonimmunogenic carriers, are found to be applied for novel drug delivery systems.^18^

The miRNAs are a kind of small endogenous single strand RNA with 18‐25 nucleotides, inducing the cleavage degradation, translation inhibition, or some other suppression on mRNAs through binding to the 3′ UTR region. And miRNAs efficiently regulate gene expression at a newly discovered level without influencing the sequence of target genes. The dysregulation of miRNAs is tightly linked with cancers and tumor microenvironment.^19^ Bioinformatics analysis suggests that miRNA‐1231 (miR‐1231) binds with the risk variant of LINC00673, conferring the susceptibility to tumorigenesis.^20^ What is more, miR‐1231 functions as a tumor suppressor with low expression and prognostic significance in glioma and gastric cancer.^21^, ^22^, ^23^ Therefore, the impact of miR‐1231 in exosomes on BM‐MSCs is obscure up to now.

The combined action of MSCs, exosomes, and miRNAs has gradually appeared.^24^, ^25^ Exosomes originated from BM‐MSCs increase CSCs population via transfer of miR‐142‐3p.^26^ While exosomal miR‐23b derived from BM‐MSCs promotes the dormancy of metastatic breast cancer cells.^27^ Our previous study found that miR‐1231 is differentially expressed in plasma exosomes of PC patients and healthy controls.^28^ Here, exosomal miR‐1231 derived from BM‐MSCs was deduced to affect PC progression. Interestingly, the low expression of miR‐1231 in peripheral blood exosomes was correlated with the TNM stage. The in vitro and in vivo experiments uncovered the inhibitory effect of exosomal miR‐1231 originated from BM‐MSCs on proliferation, cycle progression, migration, invasion, and the adhesion ability of PC cells, revealing its suppressive effect on PC. Theoretically, miR‐1231 may benefit PC diagnosis. And exosomes with high expression of miR‐1231 may develop as new and highly efficient medicines for cancer therapy, especially for PC.

## 2 MATERIALS AND METHODS

### 2.1 Cell culture

The human BM‐MSCs were isolated, identified, and preserved in our laboratory.^29^ The human PDA cell lines BxPC‐3 and MIA PaCa‐2 were purchased from Boster (Wuhan, China), and PANC‐1 and SW1990 cells were supplied by the Cell Resource Center of Shanghai Institute of Life Sciences, Chinese Academy of Sciences. The human normal pancreatic ductal epithelial cells HPDE6‐C7 were obtained from BeNa Culture Collection (Suzhou, China). BM‐MSCs and HPDE6‐C7 cells were cultured in RPMI‐1640 (Thermo Fisher Scientific) supplemented with 15% (v/v) fetal bovine serum (FBS) (Thermo Fisher Scientific), 100 units/mL penicillin (Thermo Fisher Scientific), and 100 μg/mL streptomycin (Thermo Fisher Scientific). Other PC cells were cultivated in Dulbecco's Modified Eagle's Medium (DMEM) (Thermo Fisher Scientific) supplemented with 10% (v/v) FBS. FBS used for cell culture was ultracentrifuged at 100 000 *g* for 10 hours to remove exosomes. Cells were cultured at 37°C in a humidified cell incubator with 5% CO_2_.

### 2.2 Isolation and characterization of exosomes

Exosomes were isolated according to the previous report.^30^ In brief, the supernatants of cultured cells and peripheral blood were sequentially centrifuged at 2000 *g* and 4°C for 20 minutes to remove dead cells, and were further centrifuged at 10 000 *g* and 4°C for 30 minutes to remove cell debris. The supernatants were then transferred to another clean ultra tube and ultracentrifuged at 100 000 *g* and 4°C for 70 minutes. After discarding the supernatants, the deposited exosomes were resuspended in PBS, and then ultracentrifuged at 100 000 *g* and 4°C for another 70 minutes. The purified exosomes were resuspended with PBS for further study or stored at −80°C. Immunoblots were conducted to determine the specific expression markers of exosomes, including CD63, CD9, and TSG101. The size and amount of exosomes were identified by nanoparticle tracking analysis on a Nanosight NS300 analyzer (Malvern Instruments Ltd, Malvern, UK), and the morphology of exosomes was photographed by JEM‐3010 electron microscope (JEOL).

### 2.3 Electron microscope

To observe the morphology of exosomes, 10 μL purified exosomes in PBS mixed with the same volume of 4% paraformaldehyde (PFA) was added onto the carbon film. Twenty minutes later, the carbon film was rinsed with PBStwice, two minutes each time. After absorbing the residual fluid, 10 μL of 2% uranyl‐acetate solution was added onto the carbon film to stain exosomes for 1minute. Then the film was dry in air for 5‐10 minutes. The morphology of exosomes was photographed at 200 kV on an electron microscope JEM‐3010.

### 2.4 Nanoparticle tracking analysis

Exosomes derived from BM‐MSCs were diluted with PBS at a ratio of 1:1000 to form a single particle. A quantity of 300 μL sample was injected into the Nanosight NS300 instrument with a syringe to avoid small air bubbles. Brownian motion of exosomes was observed, and size distribution, concentration, and three‐dimensional (3D) motion map of exosomes were recorded for analysis.

### 2.5 Transfection of miR‐1231 oligonucleotides and pRNAT‐U6 vector

Oligonucleotides of miR‐1231 mimics, miR‐1231 inhibitor, and the negative control (NC) were synthesized at Biomics Biotech (Nantong, China). To conduct transfection, BM‐MSCs were seeded in 6‐well plates (Corning Incorporated) at 50%‐60% confluence. After cultivation for 24 hours, cells were transfected with 50 nmol/L oligonucleotides of miR‐1231 in 250 μL Opti‐MEM^TM^ medium supplemented with 5 μL Lipofectamine^®^ 2000 (Thermo Fisher Scientific). After transfection for 48 hours, the expression change of RNAs and proteins was examined by quantitative real‐time polymerase chain reaction (qRT‐PCR) or immunoblots. To examine the consistency of transfection efficiency, 50 nmol/L miR‐1231 oligonucleotides and 1 μg pRNAT‐U6 vector (GeneScript) were co‐transfected in BM‐MSCs in 250 μL Opti‐MEM^TM^ medium supplemented with 5 μL Lipofectamine^®^ 2000 for 48 hours. Under a 100‐fold inverted fluorescence microscope, we randomly observed and counted five random visual fields of co‐transfected cells per well of a 6‐well cell culture plate, and took their mean value as the number of fluorescent cells per well and unit area. Sequences of synthesized miRNAs are listed in Table 1.

**Table 1:** Sequences of synthesized miR‐1231 oligonucleotides

Oligonucleotides	Sequences (5′ to 3ʹ)
miR‐1231 mimics	GUGUCUGGGCGGACAGCUGC
miR‐1231 inhibitor	GCAGCUGUCCGCCCAGACAC
Negative control	UCUACUCUUUCUAGGAGGUUGUGA

### 2.6 Exosome treatment

PC cells of 5 × 10^5^ were seeded in per well of a 6‐well culture plate at 50%‐60% confluence. Then, 2 μg exosomes (equivalent to those collected from about 1 × 10^6^ BM‐MSCs) resuspended in 10 μL PBS were added to PC cells in per well of a 6‐well plate. After cultivation for 24 hours, the expression level of target RNAs and proteins in cancer cells was analyzed by qRT‐PCR or immunoblots.

### 2.7 qRT‐PCR

Total RNA of cells was extracted using TRIzol (Thermo Fisher Scientific), and was treated with RNase‐free DNase (Promega) at 37°C for 1 hour to remove the genomic DNA. Reverse transcription PCR (RT‐PCR) and qPCR for determining the expression level of miR‐1231 were performed using the All‐in‐One™ miRNA qRT‐PCR Detection System (GeneCopoeia) according to manufacturer's instructions. The specific primer for miR‐1231 was GTGTCTGGGCGGACAGCTGC. The U6 gene was used as the internal control, and the specific primers was purchased from GeneCopoeia.

From January 2017 to August 2019, patients with a pathological diagnosis of PC at Hunan Cancer Hospital and Affiliated Cancer Hospital of Xiangya Medical School were recruited for this study. The peripheral blood samples were collected from 36 PC patients and 20 normal people. Exosomes in peripheral blood were extracted according to above ultracentrifugation method. Total RNA of exosomes in peripheral blood was extracted using TRIzol LS (Thermo Fisher Scientific). The expression level of miR‐1231 in exosomes was examined by qRT‐PCR.

### 2.8 Immunoblots

Cell or exosome lysates obtained with RIPA lysis buffer (Thermo Fisher Scientific) were centrifuged at 13 200 rpm and 4°C for 15 minutes, and were quantified by the BCA method. Proteins were run on a SDS‐PAGE gel according to a standard protocol and were transferred onto a PVDF membrane (Merck Millipore). The PVDF membrane was blocked with 5% skim milk, and was sequentially incubated with the primary and the second antibodies. The following antibodies were used according to the manufacturer's instructions: CD63 (CST), CD9 (CST), TSG101 (System Biosciences), EGFR (CST), Cyclin E (CST), and β‐actin (Sigma‐Aldrich Co). Protein bands on PVDF membrane were detected using the Chemiluminescent Substrate System (Thermo Fisher Scientific). β‐actin was used as the internal control.

### 2.9 MTS assay

Cells in the logarithmic phase were seeded in a 96‐well plate at a density of 2 × 10^3^ per well. After cultivation at 37°C for 24 hours in a humidified incubator with 5% CO_2_, the culture medium was changed to 100 μL conditioned medium, which contained different types of exosomes. To determine the cell viability, 10 μL of 5 mg/mL MTS solution (Promega) was added into the culture medium and incubated for three hours. Then the optical density (OD) value at 490 nm was measured using a microplate reader (Thermo Fisher Scientific). Proliferation ability of PC cells was tested at set time points. All experiments were repeated thrice.

### 2.10 Flow cytometry analysis

PC cells treated with indicated exosomes extracted from BM‐MSCs for 48 hours were collected for flow cytometry analysis. The collected cells were fixed with 70% ethanol at 4°C for 24 hours, and then were resuspended in PBS supplemented with PI Staining Solution (Dingguo Biotech) in the dark for 30 minutes. The cell cycle was analyzed on a flow cytometer EPICS XL (Beckman Coulter).

### 2.11 Wound‐healing assay

Cells in the logarithmic phase were seeded in a 6‐well plate at 50%‐60% confluence. After cultivation for 24 hours, the cells were starved with the serum‐free medium for another 24 hours. The wounds in cells were conducted using a standard 10 μL pipette tip and a ruler. Thereafter, cells were cultivated in serum‐free medium. Images of the wound‐healing process were captured every 12 hours using an inverted light microscope.

### 2.12 Transwell assay

The migration and invasion experiments were conducted using the noncoated and Matrigel‐coated 24‐well chambers with 8.0‐μm PET membrane pores (Corning Incorporated), respectively. Cells were starved in DMEM medium without FBS for 24 hours. A quantity of 600 μL culture medium with 10% (v/v) FBS as the chemoattractant was added into the lower chamber of Transwell, and 100 μL serum‐free DMEM with 1 × 10^5^ cells was added into the upper chamber. After cultivation for 24 hours, the transmigrated or invasive cells were fixed in methyl alcohol, stained with 0.2% (m/v) crystal violet (Sigma‐Aldrich Co), and rinsed in PBS (Thermo Fisher Scientific). Images of the transmigrated cells were captured under an upright light microscope. The stained cells with crystal violet were further dissolved with 500 μL of 1% (m/v) SDS. And the amount of invasive cells was qualitatively determined through measuring the OD value at 490 nm.

### 2.13 Adhesion assay

Per well of the 96‐well plate was coated with 30 μL of 40 μg/mL collagen I (Thermo Fisher Scientific) in PBS for 12 hours, and then was rinsed with PBS and dried at room temperature. After serum starvation for 8 hours, the PC cells were detached from the culture dish after incubation with 10 mmol/L EDTA for 10 minutes, and were centrifuged to remove the EDTA and resuspended at a density of 2 × 10^5^ cells/mL in DMEM supplemented with 0.1% (m/v) BSA (TaKaRa). Then 100 μL cell suspension was added into per well of the coated 96‐well plate. After cultivation at 37°C for 20 minutes, the nonadherent cells in the culture medium were discarded, and the plate was washed with 100 μL DMEM for four times. The adherent cells were further cultured in DMEM supplemented with 10% (v/v) FBS at 37°C for 4 hours, and the amount of adherent cells was determined by MTS assays.

### 2.14 Tumor xenograft and tail vein injection

The animal experiments performed in this study were approved by the Animal Care and Experiment Committee of Hunan Cancer Hospital. Every effort was made to minimize the pain of the mice. Here 24 4‐week‐old female athymic BALB/C nude mice (SJA) were fed under standard conditions at the animal care facility. To observe the effect of exosomes on tumor growth in vivo, 1 × 10^7^ BxPC‐3 cells in 200 μL sterile PBS were administered into the BALB/C nude mice by subcutaneous injection. Two weeks later, the mice were randomly separated into four groups (n = 6 each), and 100 μg exosomes originated from transfected BM‐MSCs in 100 μL PBS were administered into mice every three days through the tail vein according to previous reports.^15^, ^31^ About 3 weeks later, mice were sacrificed, and tumors were collected for further research. The tumor volume was calculated according to the following formula: tumor volume (mm^3^) = 0.5 × length × width^2^.

### 2.15 Ethics approval

This study was approved by the Institutional Review Board of Hunan Cancer Hospital. The execution of the animal experiments was approved by the Animal Care and Experiment Committee of Hunan Cancer Hospital. Human tissue samples were obtained with informed consent and were approved by the Ethics Committee of Hunan Cancer Hospital.

### 2.16 Statistical analysis

SPSS 22.0 statistical software (IBM Corporation) was used for statistical analyses. Graphs were generated using GraphPad Prism 5.0 (GraphPad Software). Student's *t* test and one‐way analysis of variance were used in the statistical analysis, and results were expressed as the mean ± standard deviation (SD) with independently biological replicates. Differences were considered statistically significant at *(*P* < .05), **(*P* < .01), and ***(*P* < .001).

## 3 RESULTS

### 3.1 Exosomes derived from peripheral blood of PC patients express low level of miR‐1231

**Figure 1:**
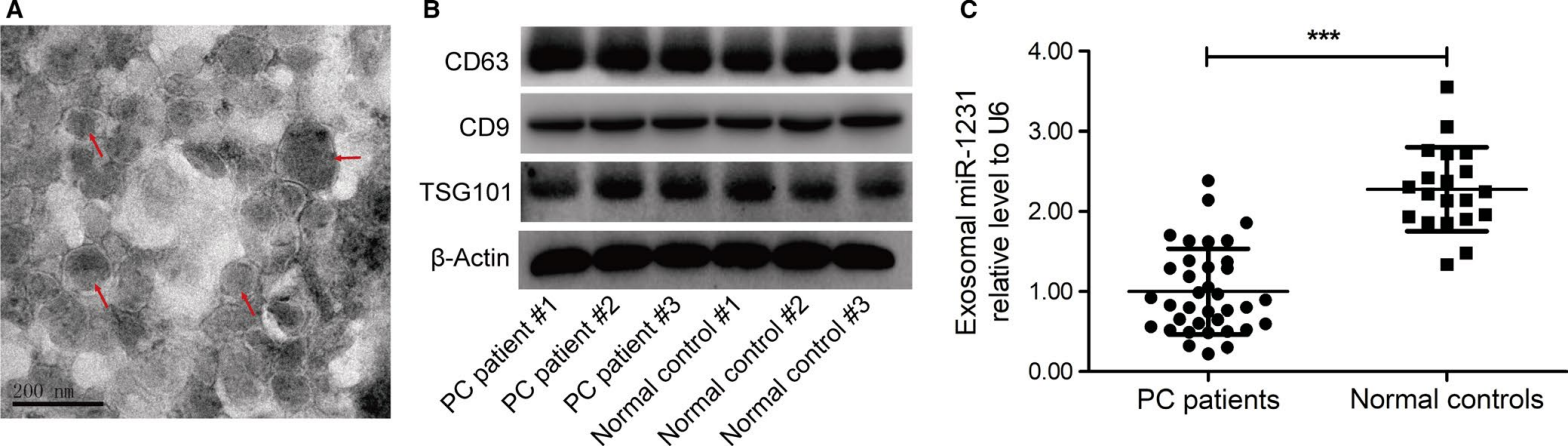
The expression level of exosomal miR‐1231 in peripheral blood of PC patients. A, Representative image for electron analysis of exosomes from peripheral blood in a PC patient. Scale bar, 200 nm. B, Immunoblots for CD63, CD9, and TSG101 in exosomes from peripheral blood of representative PC patients and normal individuals. C, Scatter plots showing the relative expression level of exosomal miR‐1231 between 36 PC patients and 20 normal individuals

To illustrate the significance of exosomal miR‐1231 in clinic, we extracted exosomes from the peripheral blood from 36 PC patients and 20 normal people. The electron analysis indicated the successful extraction of exosomes (Figure 1A), which was further approved through examining the protein expression of specific makers CD63, CD9, and TSG101 (Figure 1B). The exosomal RNAs was also extracted and expression level of miR‐1231 was quantified by qRT‐PCR. It was obvious that the expression level of miR‐1231 in exosomes derived from the peripheral blood of PC patients was significantly lower than that in normal controls (Figure 1C). Thus, miR‐1231 expression in exosomes of peripheral blood was depressed with the occurrence of PC. Moreover, no significant correlation was observed with respect to gender, age, tumor location and and history of smoking, the expression level of exosomal miR‐1231 was significantly correlated with the M stage and total TNM stage of PC (Table 2). Therefore, suppressed expression of miR‐1231 in exosomes of peripheral blood may predict PC metastasis, underlining the clinical significance of exosomal miR‐1231 research. According to these results, we speculated that the depressed expression of exosomal miR‐1231 may promote the proliferation, migration, invasiveness, and metastasis of PC.

**Table 2:** Correlation of PC clinicopathologic features and miR‐1231 expression of exosomes in peripheral blood (n = 36)

Parameters	Cases	The relative level to U6	*P*
Gender			.829
Male	21	0.983 ± 0.553	
Female	15	1.023 ± 0.527	
Age			.446
<55	13	1.092 ± 0.544	
≥55	23	0.948 ± 0.535	
History of smoking			.296
No	22	0.925 ± 0.532	
Yes	14	1.118 ± 0.536	
CA19‐9 grading			.131
Normal	14	1.170 ± 0.488	
High	22	0.892 ± 0.546	
CEA grading			.337
Normal	23	1.065 ± 0.571	
High	13	0.884 ± 0.461	
CRP grading			.710
Normal	27	1.020 ± 0.524	
High	9	0.941 ± 0.594	
Tumor location			.606
Pancreatic head	25	1.031 ± 0.525	
Pancreatic body or tail	11	0.929 ± 0.577	
T stage			.268
T1	2	1.085 ± 0.404	
T2	7	1.227 ± 0.639	
T3	10	1.138 ± 0.455	
T4	17	0.815 ± 0.524	
N stage			.113
N0	19	1.157 ± 0.511	
N1	7	0.967 ± 0.547	
N2	10	0.724 ± 0.502	
M stage			.015
M0	23	1.160 ± 0.528	
M1	13	0.717 ± 0.433	
TNM stage			.025
I	6	1.442 ± 0.535	
II	5	1.240 ± 0.445	
III	12	0.986 ± 0.525	
IV	13	0.717 ± 0.433	

### 3.2 The synthesized oligonucleotides influence the expression level of exosomal miR‐1231

**Figure 2:**
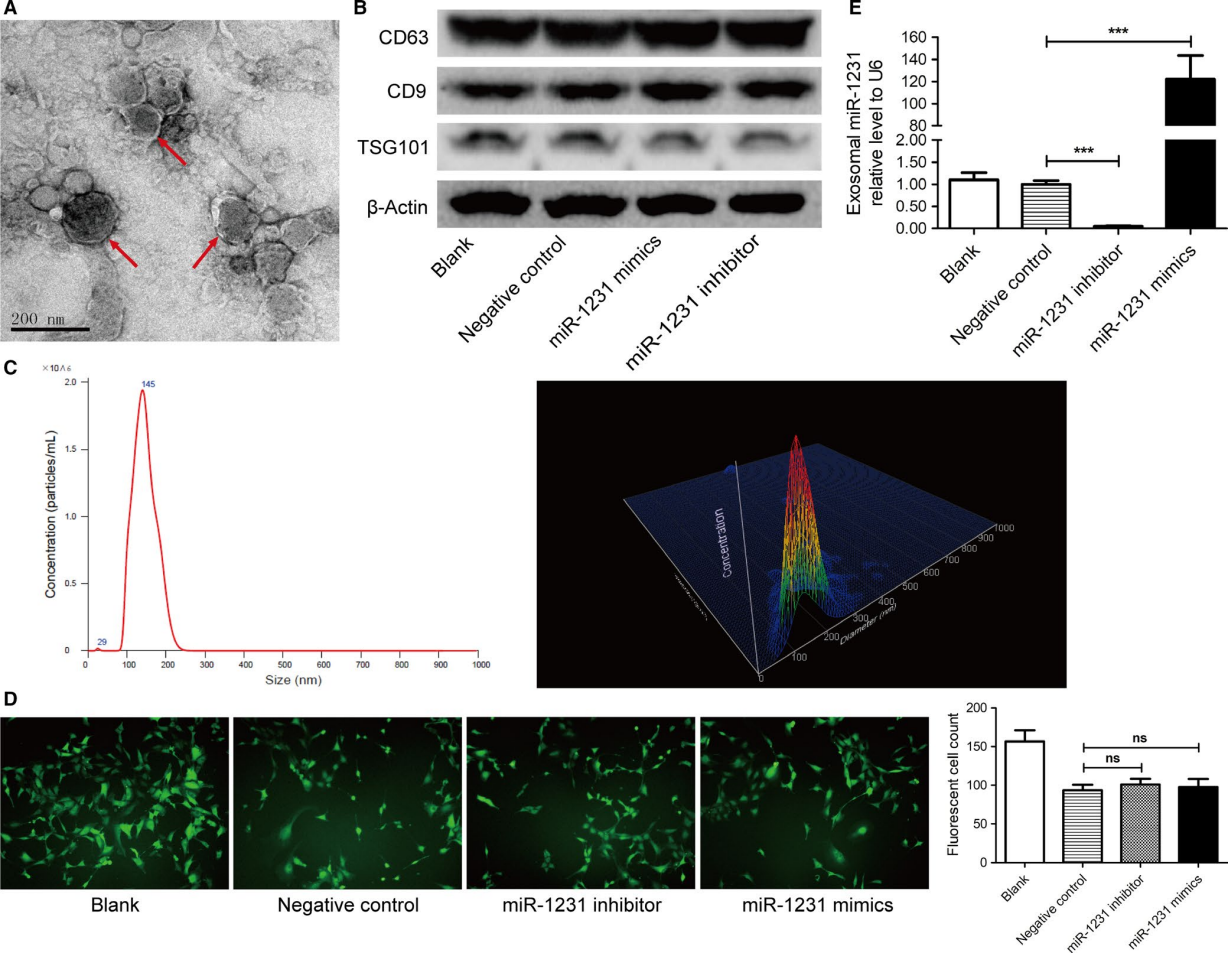
Characteristics of exosomes derived from the supernatants of BM‐MSCs. A, Representative image for electron analysis of exosomes from supernatant of BM‐MSCs. Scale bar, 200 nm. B, Immunoblots for CD63, CD9, and TSG101 in extracted exosomes derived from supernatants of BM‐MSCs that transfected with different oligonucleotides of miR‐1231. C, Brownian motion analysis for the diameter and distribution of extracted exosomes from the supernatants of BM‐MSCs. D, Representative images and statistical analysis for green fluorescence in pRNAT‐U6 vector and miR‐1231 oligonucleotides co‐transfected BM‐MSCs. E, qRT‐PCR for exosomal miR‐1231 in oligonucleotides transfected BM‐MSCs. Student's two‐sided *t* test, data are mean ± SD with three independently biological replicates (D and E). Experiments were independently replicated three times with similar results (A‐E)

BM‐MSCs are linked with cancers. Our previous research revealed the oncogenic role of exosomal miRNA‐221 derived from BM‐MSCs.^32^ Here, we deduced that exosomal miR‐1231 of BM‐MSCs might perform antitumor effect in PC. To illustrate our speculation, BM‐MSCs were transiently transfected with the synthesized oligonucleotides of miR‐1231 and the NC, and exosomes from supernatants of transfected BM‐MSCs were extracted by ultra‐centrifuging at 100 000 *g*. The canonical morphology of extracted exosomes was captured by electron microscope analysis (Figure 2A). It seems that the extracted exosomes was spherical with a diameter from 30‐150 nm. The expression of exosome markers examined by immunoblots, including CD63, CD9, and TSG101, further approved the successful isolation of exosomes in supernatants of transfected BM‐MSCs (Figure 2B). And the expression level of exosomal markers was not influenced by miR‐1231. According to the analytical results of Brownian motion, the diameter spread of exosomes was basically consistent with normal distribution (Figure 2C). In detail, the diameter of the exosomes primarily ranged from 80 to 220 nm, and the concentration of exosomes with a diameter of 145 nm was observed to have a peak value. The similar and high intensity of green fluorescence, observed in pRNAT‐U6 vector and miR‐1231 oligonucleotides co‐transfected BM‐MSCs, indicated the consistency and high efficacy of miR‐1231 oligonucleotides transfection (Figure 2D). And miR‐1231 oligonucleotides especially inhibited or promoted the expression level of miR‐1231 in exosomes from BM‐MSCs (Figure 2E). Compared with the NC group, the expression of miR‐1231 in inhibitor group was critically reduced about 95%, while that was significantly upregulated to nearly 122‐fold after transfection with miR‐1231 mimics. These results indicated that miR‐1231 expression in exosomes of BM‐MSCs can be effectively modulated through in vitro transfection.

### 3.3 Exosomal miR‐1231 originated from BM‐MSCs inhibits PC cell proliferation

**Figure 3:**
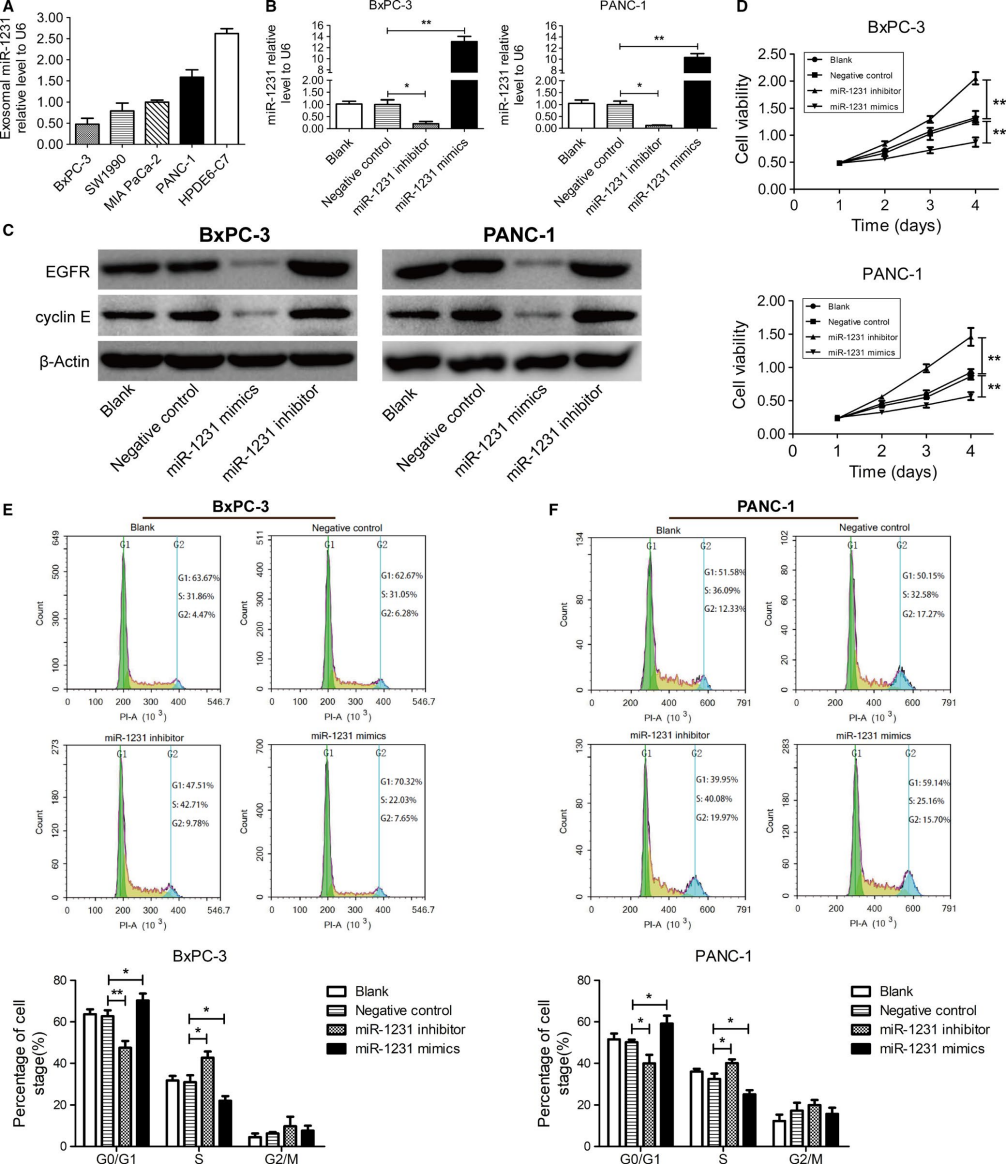
Influence of exosomal miR‐1231 derived from BM‐MSCs on proliferation and cycle progression of PC cells. A, Expression profile of exosomal miR‐1231 originated from PC cells and the pancreatic normal cells HPDE6‐7. B, qRT‐PCR for miR‐1231 expression in BxPC‐3 and PANC‐1 cells after incubation with exosomes derived from transfected BM‐MSCs with oligonucleotides miR‐1231. C, Immunoblots for EGFR and Cyclin E of BxPC‐3 and PANC‐1 cells after cultivation with exosomes derived from the supernatants of transfected BM‐MSCs with oligonucleotides miR‐1231. D, Statistical analysis for cell proliferation of BxPC‐3 and PANC‐1 examined by MTS assays. Cancer cells were cultivated with exosomes from transfected BM‐MSCs with oligonucleotides miR‐1231. E and F, Flow cytometry analysis for cell cycle progression of BxPC‐3 and PANC‐1 cells after cultivation with exosomes from supernatants of transfected BM‐MSCs with oligonucleotides miR‐1231. Student's two‐sided *t* test, data are mean ± SD with three (A, B, E and F) or four (D) independently biological replicates. Experiments were independently replicated three times with similar results (A‐F)

Next, the exosomes in the culture supernatant of PC cells and normal pancreatic epithelial cells HPDE6‐C7 were extracted, and the expression level of exosomal miR‐1231 was quantified by qRT‐PCR (Figure 3A). Compared with that of HPDEC6‐7, the expression level of miR‐1231 in exosomes secreted by PC cells was relatively lower. Among the PC cells, the expression level of miR‐1231 in PANC‐1 was the highest, while that in BxPC‐3 was the lowest. Therefore, PANC‐1 and BxPC‐3 cells were chosen for the following research and cultivated with the extracted exosomes from BM‐MSCs supernatants. Interestingly, miR‐1231 level in BxPC‐3 and PANC‐1 cells treated with exosomes from mimics transfected BM‐MSCs was increased nearly 13‐ and 10‐fold when compared with that in NC group, respectively (Figure 3B). However, the level of miR‐1231 was deceased to 20% and 12% in PC cells that cultivated with exosomes from inhibitor transfected BM‐MSCs. These results indicated that the extracted exosomes from transfected BM‐MSCs enter into PC cells, thereafter affect the expression of miR‐1231 in PC cells. Resemble with the fashion of liposome‐mediated transfection, exosomes may act like the nanocarriers entering into cells and influencing gene expression.

To further confirm the influence of exosomal miR‐1231 on PC cells, the expression level of EGFR and cyclin E, as the direct targets regulated by miR‐1231,^22^ was examined by immunoblots. Compared with that in NC group, the expression level of EGFR and cyclin E was downregulated in miR‐1231 mimics group, while upregulated in miR‐1231 inhibitor group (Figure 3C). Therefore, the expression change of exosomal miR‐1231 successfully affects gene expression in PC cells. Notably, the proliferation of BxPC‐3 and PANC‐1 cells examined by MTS was inhibited with the introduction of exosomes derived from miR‐1231 mimics transfected BM‐MSCs, while was enhanced in miR‐1231 inhibitor group (Figure 3D). Thus, exosomal miR‐1231 inhibits PC cell proliferation. The flow cytometry analysis indicated that oligonucleotides of miR‐1231 inhibitor significantly shortened the G0/G1 phase of BxPC‐3 and PANC‐1 cells (Figure 3E and F); whereas the cycle progression was significantly restrained in G0/G1 phase after cultivation with miR‐1231 overexpressed exosomes. In conclusion, the exosomal miR‐1231 inhibits the proliferation of PC cells and induces cell cycle arrest. These results implied the antitumor effect of exosomal miR‐1231, and also provided the potential of exosomes application in cancer treatment.

### 3.4 Exosomal miR‐1231 derived from BM‐MSCs inhibits migration, invasion, and adhesion to the matrix of PC cells

**Figure 4:**
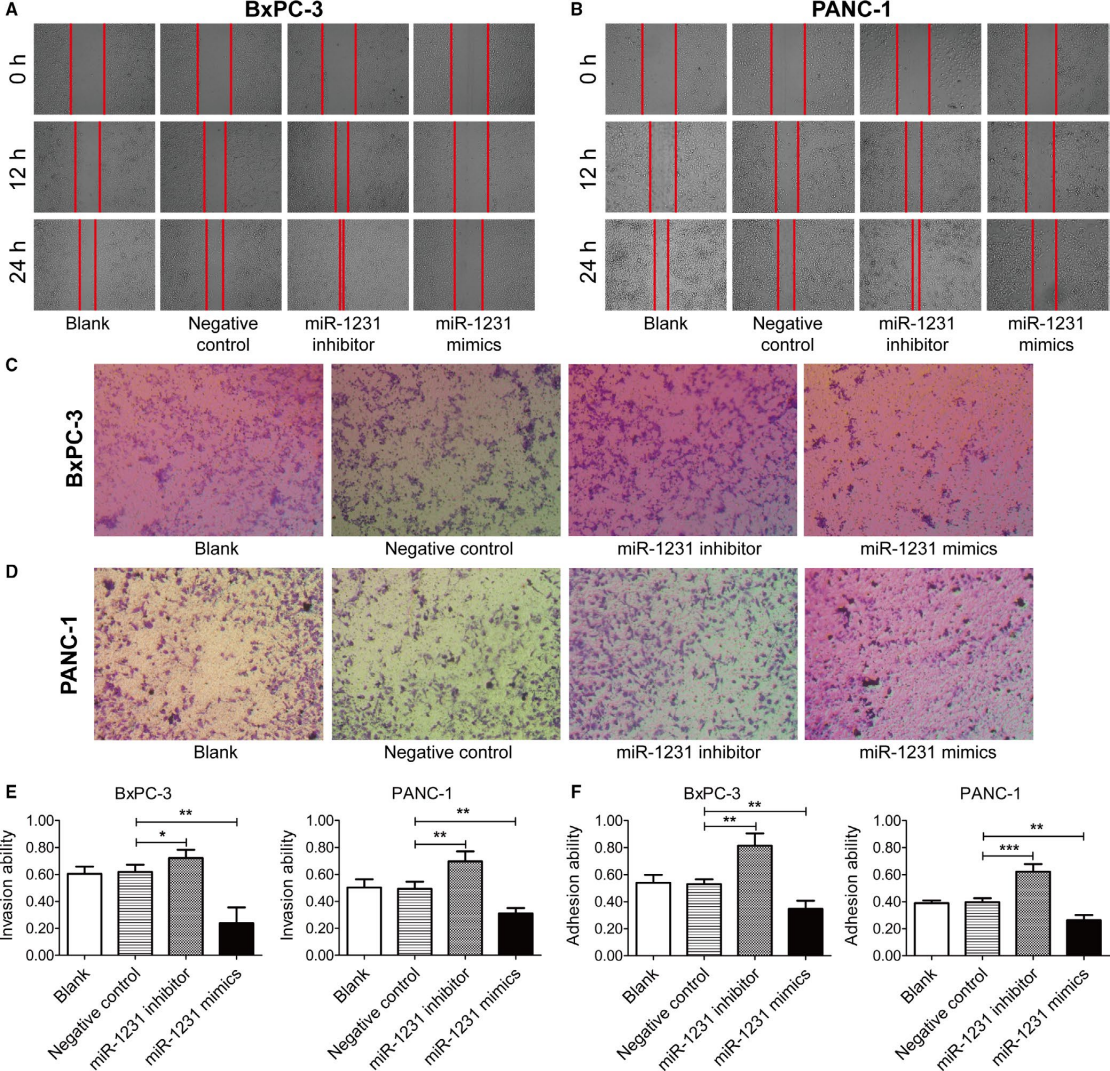
Influence of exosomal miR‐1231 on migration, invasion, and adhesion ability to the matrix of PC cells. A and B, Representative images showing the wound healing of cancer cells. BxPC‐3 and PANC‐1 cells were cultivated with exosomes derived from the supernatants of transfected BM‐MSCs with miR‐1231 oligonucleotides. C and D, Representative images exhibiting the migrated cancer cells. BxPC‐3 and PANC‐1 cells were cultivated with exosomes from the supernatants of transfected BM‐MSCs with miR‐1231 oligonucleotides. E and F, Statistical analysis for the invasion and adhesion ability of BxPC‐3 and PANC‐1 cells after cultivation with exosomes derived from transfected BM‐MSCs with miR‐1231 oligonucleotides. Student's two‐sided *t* test, data are mean ± SD with three (E) or four (F) independently biological replicates. Experiments were independently replicated three times with similar results (A‐F)

Wound‐healing assays were conducted to evaluate the regulatory effect of miR‐1231 on cell migration. Obviously, decelerated rate of wound healing of BxPC‐3 and PANC‐1 cells was appeared with the introduction of exosomes from transfected BM‐MSCs with miR‐1231 mimics (Figure 4A and B). In comparison, the rate of wound healing was accelerated in miR‐1231 inhibitor groups. These results suggested the inhibitory role of exosomal miR‐1231 in cell migration. This conclusion was further confirmed through conducting Transwell assays. Compared with that of the NC groups, the amount of the migrated BxPC‐3 and PANC‐1 cells was decreased after cultivation with exosomes from the miR‐1231 mimics‐transfected BM‐MSCs, while it increased after treatment with exosomes from miR‐1231 inhibitor‐transfected BM‐MSCs (Figure 4C and D), further approving the restriction of exosomal miR‐1231 on migration of PC cells.

We further evaluated the role of exosomal miR‐1231 in regulating cell invasion by using the Matrigel‐coated Transwell chambers. According to the statistical analysis, the invasion ability of BxPC‐3 and PANC‐1 cells was significantly suppressed in miR‐1231 mimics group, while was markedly upregulated in miR‐1231 inhibitor group (Figure 4E). Thus, the extracted exosomes from supernatants of transfected BM‐MSCs influences the invasion ability of PC cells, and exosomal miR‐1231 restrains the invasiveness of PC cells.

The regulatory role of exosomal miR‐1231 in the adhesion ability of PC cells to the matrix was examined in study. As the statistical graph shown, 1.5‐fold of PANC‐1 cells cultured with exosomes derived from supernatants of miR‐1231 inhibitor‐transfected BM‐MSCs adhered to the matrix (Figure 4F). Similar results were also observed in BxPC‐3 cells. However, the number of adherent PC cells was significantly decreased after cultivation with exosomes in miR‐1231 mimics group. Thus, exosomes with high expression of miR‐1231 effectively inhibit the adhesion ability of PC cells to the matrix.

In conclusion, exosomal miR‐1231 performs the antitumor effect through inhibiting the proliferation, migration, invasion, and the adhesion ability to the matrix of PC cells. The upregulated cancer characteristics of PC cells triggered by exosomes from the supernatants of miR‐1231 inhibitor‐transfected BM‐MSCs may be a new mechanism to explain the frequent metastasis of PC in clinic. And exosomes with a high expression level of miR‐1231 may be a potential therapeutic medicine in cancer treatment, especially for PC.

### 3.5 Exosomal miR‐1231 derived from BM‐MSCs suppresses tumor growth in vivo

**Figure 5:**
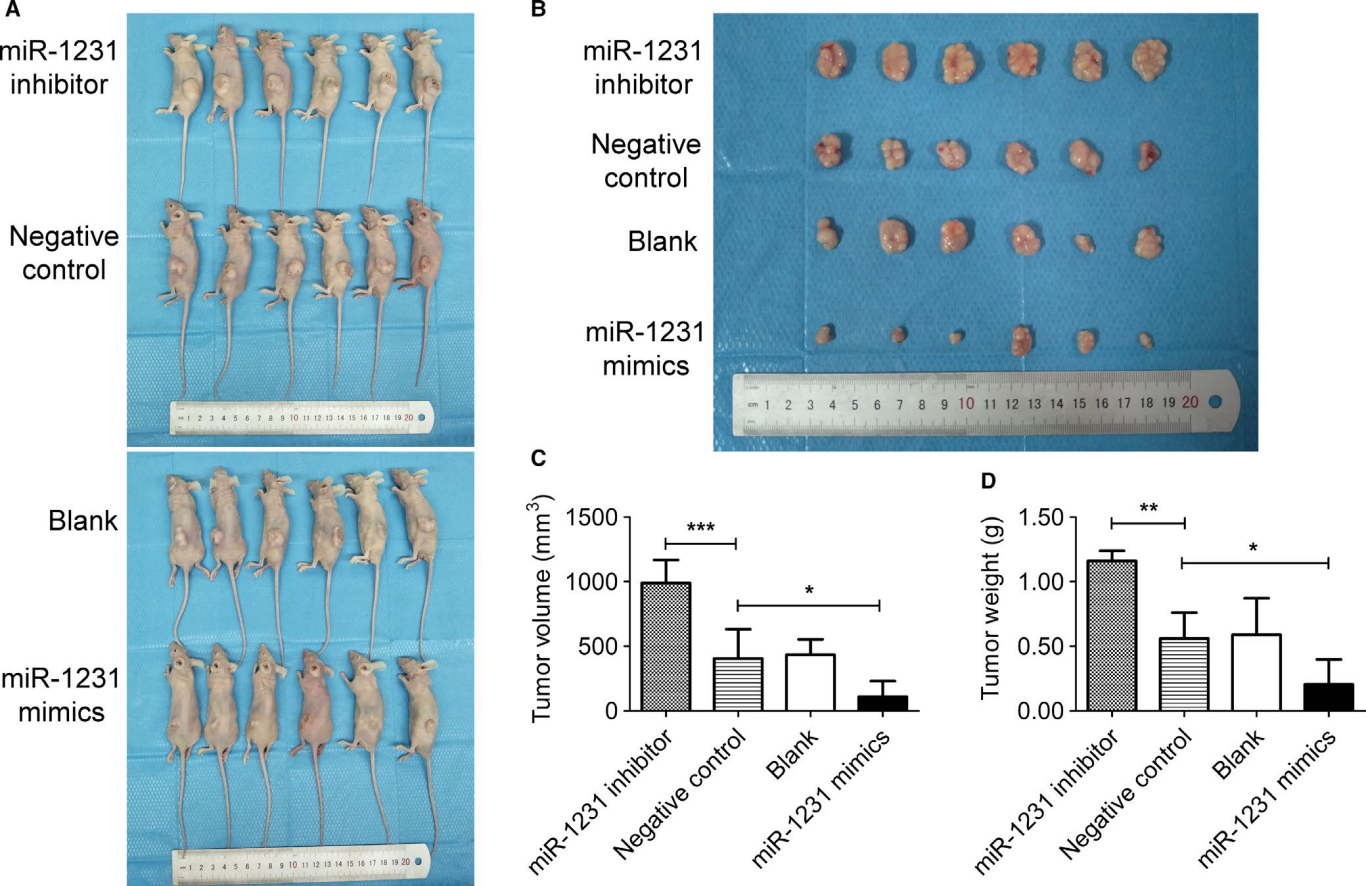
Exosomes derived from BM‐MSCs with overexpressed miR‐1231 suppresses tumor growth in vivo. A, Image showing the formed tumors of BxPC‐3 PC cells in mice after tail vein injection with different exosomes. Exosomes were extracted from the supernatants of BM‐MSCs that transfected with different miR‐1231oligonucleotides. B, Image showing the formed tumors after striping from the mice bodies. The minimum scale of the stainless steel ruler is 1 mm. C and D, Statistical analysis for tumor volume and weight in B. Student's two‐sided *t* test, data are mean ± SD with six independently biological replicates (C and D)

To evaluate the therapeutic potential of miR‐1231 overexpressed exosomes, BxPC‐3 cells were subcutaneously injected into female BALB/C nude mice. After tumor formation in vivo, exosomes extracted from BM‐MSCs transfected with miR‐1231 oligonucleotides were administered into mice by tail vein injection. As a result, the injected exosomes influenced the growth of tumors (Figure 5A). Compared with that in the NC group, the size of tumors was smaller in the miR‐1231 mimics group, while it was was larger in the miR‐1231 inhibitor group (Figure 5B). The statistical analysis of the tumor volume and weight further confirmed our observation that tumor growth was dramatically inhibited by exosomes extracted from supernatants of miR‐1231 mimics transfected BM‐MSCs (Figure 5C and D). However, treatment with exosomes from the miR‐1231 inhibitor group significantly promoted tumor growth. Therefore, the growth rate of formed tumors was suppressed by the administration of exosomes with a high expression of miR‐1231 derived from BM‐MSCs, thus confirming the therapeutic potential of exosomes in clinic and the vital effect of exosomal miR‐1231 on controlling PC development. These findings further provided reasonable explanation for the low level of exosomal miR‐1231 in peripheral blood of PC patients.

## 4 DISCCUSSION

With the rapid development of current medicine, more and more diseases have been successfully cured in clinic. However, limited solutions are efficient in cancer therapy. Exosomes in supernatants of cultivated cells and peripheral blood, as a kind of extracellular vesicles with a diameter from 40 to 200 nm, influence metastasis,^33^ therapy and drug resistance,^34^, ^35^ angiogenesis,^36^ epithelial mesenchymal transition (EMT) of cancers, and tumor environment.^37^, ^38^ In this study, a low expression of exosomal miR‐1231 was found in peripheral blood of PC patients. Notably, the expression level of miR‐1231 in exosomes was significantly correlated with the TNM stage of PC in clinic. The reduced expression of exosomal miR‐1231 in peripheral blood may indicate cancer metastasis, suggesting the critical regulatory role of exosomal miR‐1231 in cancer.

Exosome‐mediated transfer of microRNAs is a novel mechanism of genetic exchange between cells.^39^ And among various prognostic, diagnostic, and therapeutic biomarkers, miRNA has emerged as a powerful biomarker for the detection, treatment, and monitoring of response to therapy in cancer.^40^ Especially, exosomes provide shuttle cargos for transferring microRNA from cell‐to‐cell and leading to abnormal expression of genes, resulting in tumorigenesis and cancer development.^41^, ^42^ For example, natural killer‐derived exosomal miR‐186 inhibits neuroblastoma growth and immune escape.^43^ In our study, exosomes with differential expression of miR‐1231 extracted from the supernatant of BM‐MSCs influenced the endogenous miR‐1231 expression of PC cells. Regulation on proliferation, migration, invasion, cell‐cycle progression, and tumor growth in vivo with the treatment of exosomes suggested the inhibitory effect of exosomal miR‐1231 on PC, underlining the high efficiency of exosomes in gene information exchange.

The synthesized nanoparticles have been regarded as a potential solution for developing specific medicine targeting neoplasm. However, the disadvantage of nanoparticles in degradation in vivo limits the clinical application. As the nontoxic and nonimmunogenic carriers, exosomes can be modified and developed as new and highly efficient drugs for cancer patients from perspectives of tumor cells, microenvironment, or immunity.^44^, ^45^, ^46^ The bulk, safe, and cost‐effective production of exosomes can be obtained from bovine milk.^47^ Notably, exosomes derived from MSCs enhance radiotherapy‐induced cell death in tumor and metastatic tumor foci.^48^ Especially, exosomal miRNAs are highly suitable candidates for use as biomarkers in personalized cancer medicine.^49^ Actually, stem cell‐secreted exosomes orchestrate various autocrine and paracrine functions which alter tumor microenvironment, growth and progression.^50^ And MSCs accumulating at tumor sites increased with radiotherapy are attractive for directed cancer therapy.^51^ By conducting the peripheral blood experiments of patients and healthy people, we speculated that BM‐MSCs may be one of the sources of serum exosomal miR‐1231. Our in vitro and in vivo experiments verified that the impact of BM‐MSCs on PC may be realized through the secretion of exosomal miRNA‐1231. Our previous research confirms that different PC cells express a small amount of miR‐1231, and a very small amount of exosomal miRNA‐1231 can be detected in the supernatant of PC cells.^28^ Compared with the normal pancreatic ductal epithelial cells, the exosomal miRNA‐1231 content in supernatant of PC cells is much lower. Therefore, the effect of exosomal miRNA‐1231 secreted by PC cells is almost negligible. To further exclude the influence of exosomal miR‐1231 from PC cells themselves, we chose two PC cell lines with relatively minimum miRNA‐1231 content (BxPC‐3) and relatively maximum miRNA‐1231 content (PANC‐1) for follow‐up experiments based on Figure 3A. Our study focused on and uncovered the potential value of miR‐1231 overexpressed exosomes in PC therapy. Whereas, owing to the limitations of the experimental conditions, we failed to complete the bone marrow depletion test for vigorous in vivo research. So some meaningful work are still worthy to be conducted in future study.

The innovations and significance of our study were mainly embodied in the following. First, the low expression level of exosomal miR‐1231 in peripheral blood was found to be significantly correlated with the TNM stage of PC, thus revealing the inhibitory role of exosomal miR‐1231 in tumor growth and cancer metastasis. Second, in vitro and in vivo experiments revealed the suppression of exosomal miR‐1231 derived from BM‐MSCs on restricting the characteristics of PC. In conclusion, miR‐1231 may be an important indicator for PC diagnosis in clinic. Exosomes with high level of miR‐1231 may develop as promising medicines for PC therapy in the future.

## CONFLICT OF INTEREST

The authors have declared that there are no conflicts of interest.

## Data Availability

All data of this article are available.
